# The homing and inhibiting effects of hNSCs-BMP4 on human glioma stem cells

**DOI:** 10.18632/oncotarget.7472

**Published:** 2016-02-18

**Authors:** Shuang Liu, Feng Yin, Mingming Zhao, Chunhui Zhou, Junlin Ren, Qiming Huang, Zhongming Zhao, Ramkrishna Mitra, Wenhong Fan, Ming Fan

**Affiliations:** ^1^ Department of Neurosurgery, Navy General Hospital, PLA, Beijing 100048, China; ^2^ Department of Brain Protection & Plasticity Research, Beijing Institute of Basic Medical Sciences, Beijing 100850, China; ^3^ Department of Biomedical Informatics, Vanderbilt University Medical Center, Nashville, TN 37203, USA; ^4^ Departments of Psychiatry and Cancer Biology, Vanderbilt University Medical Center, Nashville, TN 37232, USA; ^5^ Center for Precision Health, School of Biomedical Informatics, University of Texas Health Science Center at Houston, Houston, TX 77030, USA; ^6^ National Institutes for Food and Drug Control, Beijing 100050, China

**Keywords:** human glioma stem cells, human neural stem cells, BMP4, homing effects, inhibiting effects

## Abstract

Malignant gliomas patients have a poor survival rate, partially due to the inability in delivering therapeutic agents to the tumors, especially to the metastasis of human glioma stem cells (hGSCs). To explore whether the human neural stem cells (hNSCs) with an over-expression of BMP4 (hNSCs-BMP4) can trace and inhibit hGSCs, in this study, we examined the migration of hNSCs to hGSCs using transwell assay *in vitro* and performed the fluorescent tracer experiment *in vivo*. We examined the proliferation, differentiation, apoptosis and migration of hGSCs after co-culturing with hNSCs-BMP4 *in vitro* and tested the tropism and antitumor effects of hNSCs-BMP4 in the established brain xenograft models of hGSCs. We found that hNSCs-BMP4 could secrete BMP4 and trace hGSCs both *in vitro* and *in vivo*. When compared to the normal human astrocytes (NHAs) and hNSCs, hNSCs-BMP4 could significantly inhibit the invasive growth of hGSCs, promote their differentiation and apoptosis by activating Smad1/5/8 signaling, and prolong the survival time of the tumor-bearing nude mice. Collectively, this study suggested that hNSCs-BMP4 may help in developing therapeutic approaches for the treatment of human malignant gliomas.

## INTRODUCTION

Glioma is the most common malignant brain tumor in the central nerve system (CNS). Some of its forms, especially glioblastoma (GBM), are often resist current treatments and recur frequently. Surgical excision, radiation and chemotherapy do not improve the prognosis of the malignant gliomas, as the mean survival period for GBM does not exceed 16 months [1, 2]. Eliminating glioma cells by conventional therapy is hindered by the exceptional infiltration of the glioma cells into the surrounding neural tissues. These infiltrated tumor cells act as seeds for recurrent tumor growth [3]. Recently, it has been reported that human glioma stem cells (hGSCs) are the main reason for the origin, invasion and resistance of GBM to radio and chemotherapy Therefore, new therapeutic strategies are needed to attack GSCs in the main tumor mass and the metastatic tumor nests [4-6]. Gene therapy appears to be an ideal candidate approach for brain tumor therapy, because of its selective toxicity to tumor cells [7]. However, there has been no significant advance in gene therapy for brain tumors due to the major limiting factors in the delivery system. For instance, commonly used vectors have little or no migratory potential or specific tropism. This limits the effectiveness of gene therapy to reach into the tumor nest and glioma cells that have infiltrated the normal brain parenchyma [8]. Indeed, it has been proven that intratumoral injection of viral vectors cannot deliver therapeutic genes to many solid tumor cells [9]. Systemic intravenous or intra-arterial administration of antibodies has been limited by the neutralizing effects of antibodies and immune-mediated organ toxicity [10, 11]. Therefore, there long has been a need to develop an effective and safe vehicle to deliver therapeutic molecules to the areas of glioma cell invasion. Many *in vivo* and *in vitro* studies have demonstrated that human neural stem cells (hNSCs) have a unique capacity to migrate throughout the brain and to chase invading tumor cells, such as glioma, which indicates their therapeutic potential [12-14]. Aboody et al. have shown that intracranial injection of NSCs that have a tropism for brain tumors could be exploited therapeutically [15]. Similarly, Ehtesham et al. have shown that locally-injected NSCs that were engineered to deliver interleukin-12 or tumor necrosis factor–related apoptosis inducing ligand (TRAIL) could slow the growth of brain tumors [16, 17]. These studies have convinced investigators that the NSCs that express therapeutic genes can be stably engrafted in brain and chase tumor cells.

Bone morphogenetic proteins (BMPs) are a family of cytokines that have complex effects on neural stem and progenitor cells. In NSCs that are derived from early embryos, BMPs appear to promote proliferation and neuronal differentiation mediated by BMPR-IA. In contrast, NSCs that are derived from adult brains undergo astrocytic differentiation in response to BMPs mediated by BMPR-IB [18, 19].

Our previous studies have shown that overexpression of BMPR-IB can arrest the growth of glioblastoma cells in which there were almost no expression of BMPR-IB and bring about their differentiation by the activation of Smad1 and up-regulation of p21 and p27kip1 *in vitro* and *in vivo* [20, 21]. The pro-differentiated role of BMPs/Smad1 in NSCs and glioblastoma cell lines has inspired investigators to further study their roles in hGSCs. Piccirillo et al. reported that treatment of GBM-derived brain tumor stem cells (BTSCs) with BMP4 had the strongest effect in inhibiting the proliferation of BTSCs, inducing their differentiation and, reducing their ability to form tumors in immune-deficient mice [22]. Thus, these BTSCs behaved like “older” NSCs in their response to BMPs. In this matter, Lee and colleagues also found that BMPs promoted apparent glial differentiation in BTSCs in some patient-derived samples [23]. In the present study, we used hNSCs as a vehicle for delivery of BMP4 to GBM in order to develop a novel and effective mean to trace and eliminate hGSCs.

## RESULTS

### Isolation and characterization of hNSCs and hGSCs

**Figure 1:**
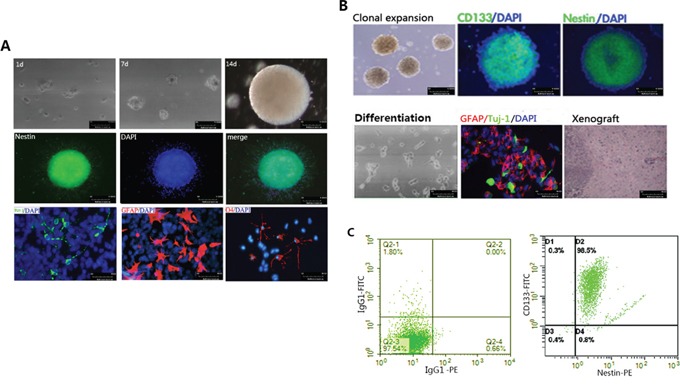
Characterization of hNSCs and hGSCs **A.** hNSCs characterization. Upper panel: Phase-contrast photograph showing neurospheres grown in serum-free media for 1-14days. (Scale bar, 100μm). Middle panel: Cells in the neurosphere are immuno-positive for nestin, (scale bar, 100μm). Lower panel: After differentiation for two weeks, the differentiated cells were stained against markers for neurons (Tuj1), astrocytes (GFAP) and oligodentrocytes (O4), (scale bar, 50μm). **B.** Upper panel: After culturing *in vitro* for seven days, the hGSCs formed neurosphere. We stained tumor spheres with mouse antibody against human CD133 and nestin. Most tumor cells in the sphere were CD133 and Nestin positive (Green) (scale bar, 50μm.). Lower panel: The spheres of hGSCs were transferred to poly-D-lysine coated chamber slides and cultured in DMEM-F12 that was supplemented with 10% FBS. After 24 hours of culturing, the tumor spheres began to adhere and differentiate. After differentiation, GFAP immunoreactive positive astrocytes (Red) and Tuj1 immunoreactive positive neurons (Green) were observed (scale bar, 50μm.). One month after the intracranial transplantation of 1×10^8^ hGSCs, H&E staining showed that the hGSCs formed invasive neoplasm in the nude mice (scale bar 50μm). **C.** FACS analysis showed that the proportion of CD133 and nestin double positive cells were more than 90% (Right panel). Left panel: isotype control.

Human NSCs were cultured *in vitro* by the previously described procedures [24]. After being cultured for one to two weeks *in vitro*, hNSCs formed small spheres that exhibited the morphological properties that are typical of neurospheres (Figure 1A, upper). These NSCs expressed a high level of early neuroectodermal marker, Nestin (Figure 1A, middle). Differentiation of hNSCs was initiated after three days by removing mitogens, plating the cells onto poly-L-lysine, and adding 10% FBS. Two weeks later, these cells had acquired the morphologic and phenotypic characteristics of neurons (Tuj-1 positive), astrocytes (GFAP positive) and oligodentrocytes (O4 positive) (Figure 1A, lower).

Human GSCs were isolated and cultured as described in the Materials and Methods. After one week of primary culturing, we obtained tumor spheres from the GBM tissues. These tumor spheres possessed the ability of clonal expansion (Figure 1B). Immunofluorescence and FACS analysis showed that most of the cells in the tumor spheres were double positive on CD133 and Nestin (Figure 1C). When the tumor cells derived from these tumor spheres were differentiated, GFAP positive astrocytes and Tuj-1 positive neurons were detected, although the morphology of these cells still remained immature (Figure 1B). After being implanted intracranially, these cells may develop brain tumors (Figure 1B). Together, these data indicate that the CD133 and nestin double positive cells in the tumorspheres are GSCs.

### The stable NSCs-BMP4/RFP cell line expresses BMP4 in an exocrine manner

**Figure 2:**
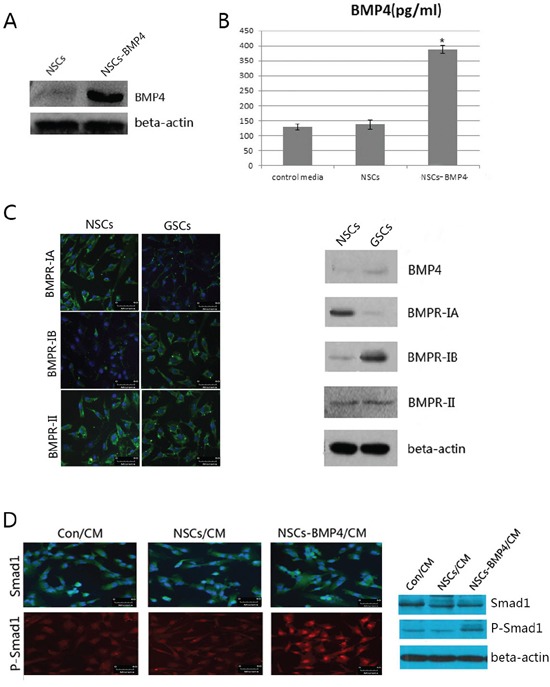
NSCs-BMP4-RFP could secret BMP4 and induce phosphorylation of Smad1 of hGSCs **A.** Expression of BMP4 in the NSCs and NSCs-BMP4 cell lines was determined by western blot assay. **B.** Production of BMP4 in supernatants of 24 hours cultures of NHAs, NSCs and NSCs-BMP4 cells were measured by ELISA. Histograms show the levels of BMP4. (*p < 0.05, Student's t-test). **C.** The expressions of BMPs/BMPRs signaling molecules in hNSCs and hGSCs. Left panel: Immunofluorescent staining for the expressions of BMPR-IA, BMPR-IB and BMPR-II (scale bar 50μm)). Right panel: Western blot analyzed the expression of BMP4 and BMP receptors. **D.** The activation of Smad1 protein in hGSCs after addition of Con/CM, hNSCs/CM or hNSCs-BMP4/CM. Left panel: Immunofluorescent staining for phospho-Smad1(P-Smad1). After 48 hours of culturing supernatants addition, the P-Smad1 was significantly greater and accumulated to the nuclei of hGSCs, which be added with the culture supernatant of NSCs-BMP4 (scale bar 50μm)). Right panel: The expressions of Smad1 and P-Smad1 in hGSCs after the addition of different CM detected by WB.

The infection of NSCs with a lentivirus that contains the RFP and BMP4 sequences generated the stable NSCs-BMP4/RFP cell line. Western blot assay showed a greater BMP4 expression in the NSCs-BMP4/RFP cell line than in NSCs (Figure 2A). Furthermore, we detected BMP4 secretion in the culture media by ELISA assay, and found that BMP4 in the CM of NSCs-BMP4/RFP increased significantly in comparison to the control group (Figure 2B).

### The expressions of BMP receptors in hGSCs

To study the effects of BMP4 on hGSCs, we examined the expression of BMP receptor subtypes, namely, BMPR-IA, BMPR-IB, and BMPR-II in normal hNSCs and hGSCs. The results of immunofluorescence staining and western blot analysis showed that BMPR-II was present in both hNSCs and hGSCs. However, BMPR-IA and BMPR-IB were expressed specifically in hNSCs and hGSCs, respectively (Figure 2C). This result is supported by the previous reports, which suggested that BMPR-IA and BMPR-IB may have different effects on BMPs signaling [25, 26]. Thus, we hypothesize that BMPs signaling may cause hNSCs and hGSCs to act differently.

### The activation of smad1 proteins in hGSCs

It is known that the canonical BMPs signaling pathway utilizes intracellular Smad proteins [20]. Thus, we conducted western blot and immunofluorescence assays to examine the expression and location of Smad1 and phospho-Smad1 proteins after co-culturing with a control condition medium (Con/CM), condition media (CM) of NSCs (NSCs/CM) and of NSCs-RFP/BMP4 (NSCs-BMP4/CM), respectively. The results showed that after addition of NSCs-BMP4/CM at the 48th huor, the expression of phospho-Smad1 had increased and the latter accumulated in the nucleus of hGSCs (Figure 2D).

### BMP4 loaded hNSC induced differentiation and apoptosis, and inhibited proliferation and migration of hGSCs by activating the BMP/Smad1 signaling pathway

**Figure 3:**
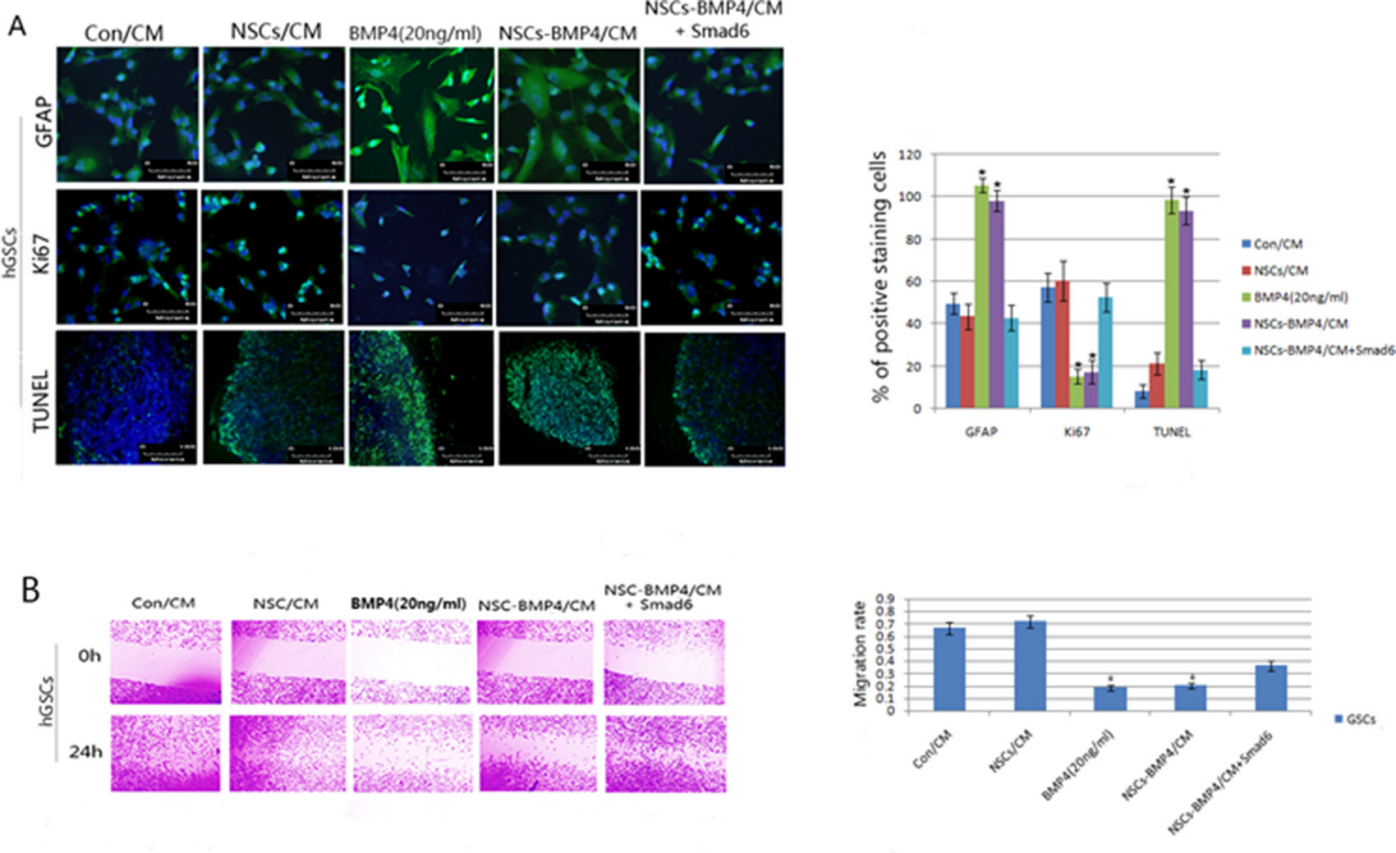
BMP4 induces differentiation and apoptosis, and also inhibits proliferation and migration of hGSCs Human GSCs were initially expanded in serum-free culture media, followed by the addition of Con/CM, NSCs/CM, BMP4 (20ng/ml), NSCs-BMP4/CM and NSCs-BMP4/CM +Smad6. **A.** Expressions of GFAP and Ki67 were examined by immunofluorescence. Apoptotic cells were determined by TUNEL assay (left panel). The percentages of GFAP, Ki67 and TUNEL-positive cells were analyzed by Student's t-test. Bars are means ± SD (right panel, *p < 0.05). **B.** Left panel: Wound scraping assay results showed that the distance of migration of hGSCs were significantly lower after being co-cultured with BMP4 (20ng/ml) and NSCs-BMP4/CM for 24 hours, and that Smad6 transfection could increase the migration distances of the hGSCs that were co-cultured with NSCs-BMP4/CM. Right panel: All experiments were conducted in triplicate in three independent sets. The values are shown as means ± SD, *p < 0.05).

In order to determine the effects of hNSCs-BMP4 on hGSCs growth *in vitro*, Con/CM, NSCs/CM, BMP4 (20ng/ml), and NSCs-BMP4/CM were applied to hGSCs for 72 hours. We found that only BMP4 and NSCs-BMP4/CM would increase the expression of GFAP and induce adherence and neurite outgrowth of hGSCs (Figure 3A). We also determined whether NSCs-BMP4/CM could induce apoptosis of hGSCs using the TUNEL assay. We observed after 72 hours following the administration of NSCs-BMP4/CM that apoptosis of hGSCs was induced by 75% and 65% compared to the use of Con/CM and NSCs/CM, respectively. Moreover, the apoptosis that was induced by NSCs-BMP4/CM was comparable with that induced by positive control-BMP4 (20ng/ml) (Figure 3A, *P* < 0.05, student's t-test).

Consistently, Ki67 staining in hGSCs, cultured with BMP4 (20ng/ml) and NSCs-BMP4/CM, was reduced significantly then it was cultured with Con/CM and NSCs/CM groups (Figure 3A, *P* < 0.05, student's t-test). Next, we examined the effect of NSCs-BMP4/CM on glioma cell migration using the wound-scraping assay. As indicated in Figure 3B, the hGSCs that co-cultured with BMP4(20ng/ml) and NSCs-BMP4/CM exhibited considerably slower migration and decreased cell spreading within 24 hours than hGSCs that were co-cultured with con/CM and NSCs/CM. It may be noted that Smad6 is the specific inhibitor of the BMPs/Smad1 signaling pathway. We observed that the effects of NSCs-BMP4/CM on hGSCs were blocked by co-expression of Smad6 without interfering with receptor-mediated phosphorylation of Smad1 (Figures 3A, 3B and Supplementary Figure S1). These results indicate that hGSCs are capable of responding to BMP4 by phosphorylation of Smad1 and subsequent activation of BMPs/Smad1 signaling pathway.

### hNSCs migrate toward hGSCs *in vitro* and *in vivo*

**Figure 4:**
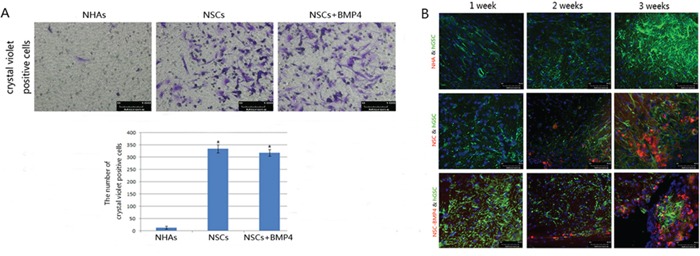
NSCs migrate to hGSCs *in vitro* and *in vivo* **A.** Upper panel: Transwell assay showed that the numbers of NSCs and NSCs-BMP4 that migrated through a micropore membrane were significantly higher than NHAs. Lower panel: Student's t-test was used to analyze the number of migration cells. All experiments were performed in triplicate in three independent sets. The values are shown as mean ± SD, *p < 0.05. **B.** After intracranial implantation of NHAs, NSCs and NSCs-BMP4 for 1, 2 and 3 weeks, the representative images of fluorescence microscopy showed tropism of NHAs-RFP, NSCs-RFP and NSCs-BMP4-RFP to intracerebral tumors of nude mice (scale bar 50μm). Tumor cells are shown in green and hNSCs appear in red.

Using an *in vitro* migration chamber, we observed that both NSCs-BMP4 and NSCs migrated in response to CM from hGSCs, whereas NHAs (negative control) migrated very little (*P* < 0.05, Student's test) (Figure 4A). To determine if hNSCs or BMP4 loaded hNSCs would migrate in response to glioma *in vivo*, we assayed *in vivo* migration using a two-color fluorescence labeling approach. We used hGSCs that constitutively expressed green fluorescent protein (GSCs-GFP) and hNSCs that constitutively expressed the mCherry red fluorescent protein (NSCs-RFP). After intracranial implantation of NHAs, NSCs and NSCs-BMP4 for 1 week, 2 weeks and 3 weeks, the brains of the nude mice bearing xenografted gliomas were sectioned and examined under fluorescence microscopy. We observed a clear and detailed anatomic representation of specific hNSCs migration in response to glioma pathophysiology (Figure 4B). After NHAs-RFP, NSCs-RFP and NSCs-BMP4-RFP were injected into the hemisphere directly contralateral to the GSC-GFP injection site for 14 days, we observed specific and appreciable engraftments of NSCs-BMP4-RFP and NSCs-RFP into malignant glioma. After 21 days, we also observed that the xenografted sites were almost full of NSCs-BMP4-RFP and NSCs-RFP (Figure 4B). However, we did not find NHAs-RFP in the GSC-GFP injection site until the end of one month (Figure 4B).

### NSCs loaded with BMP4 induced differentiation and apoptosis, and reduced invasive growth of xenografted glioma *in vivo*

**Figure 5:**
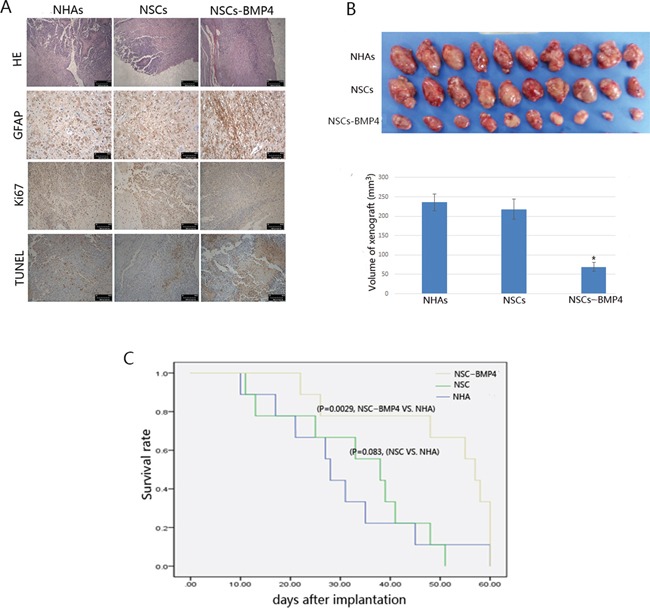
BMP4-loaded hNSCs inhibit the growth of xenografted glioma *in vivo* **A.** H&E staining analysis showed that NSCs-BMP4 could significantly inhibit the invasion of intracranial glioblastoma. The immunohischemical (IHC) analysis showed that NSCs-BMP4 effectively increased the expression of GFAP, and down-regulated the expression of Ki67 of xenografts. A TUNEL assay in xenograft tumor sections revealed that NSCs-BMP4 increased the apoptosis of intracranial glioblastomas. **B.** Upper panel: BMP4-loaded hNSCs suppresses the growth of subcutaneous xenograft of hGSCs. The tumor mass that was raised from hGSCs that were incubated with NSCs-BMP4 was significant smaller than those that were incubated with NHAs and NSCs. Lower panel: The volume of tumors of the groups was mentioned above. Bars are means ± SD. *p < 0.001, n=10 per group. **C.** BMP4-loaded hNSCs improves the survival of nude mice that have been implanted with hGSCs. Kaplan-Meier survival curves and log-rank analysis of three experimental groups were completed. The groups consisted of (1) nude mice that were inoculated with hGSCs and NHAs as a control group (n=10); (2) nude mice that were inoculated with hGSCs and hNSCs (n=10), P=0.083 versus a control group; and (3) nude mice that were inoculated with hGSCs and NSCs-BMP4 (n=10), P=0.00029 versus a control group.

Hematoxylin and Eosin (H&E) staining showed that hNSC-BMP4 could significantly inhibit the infiltration of the xenografted gliomas in the nude mice brain. Immunohistochemical staining and TUNEL assays showed that there were more GFAP-positive hGSCs and TUNEL-positive hGSCs, and fewer ki67-positive hGSCs in the brains that were inoculated with hGSCs and hNSC-BMP4, than those in the NHA and NSCs groups (Figure 5A). Next, we conducted another *in vivo* study using athymic mice that had received subcutaneous (s.c.) flank injections of hGSCs. As shown in Figure 5B, mice that received s.c. injections of hGSCs, co-cultured with CM of hNSC-BMP4, showed an overall decrease in tumor volume (69.4±16.5mm^3^) in comparison to mice that had received an s.c. injection of hGSCs co-cultured with CM of hNSC (218.1±22.7mm^3^) or CM of NHAs (235.9±63.3mm^3^). These results indicated that the NSCs that produced BMP4 were able to induce differentiation and apoptosis of glioma cells and to reduce the invasion and volume of the xenografted tumors.

### BMP4 loaded NSCs improved overall survival of GBM-affected mice

To examine further the effect of BMP4 on the survival of nude mice that have GBM, we constructed Kaplan-Meier survival curves. We had three experimental groups in this experiment, with 10 tumor-bearing nude mice in each group. The three groups were inoculated with NHAs, hNSCs, and hNSCs-BMP4, respectively. We followed the protocol of implantation and treatment as described previously [24] and the mice received no additional treatment. The time of death (in days) was recorded for each subject. We found that the mice that received hNSCs-BMP4 survived for a significant longer time than those that were inoculated with NHAs group (the log-rank test *P* < 0.005) (Figure 5C).

## DISCUSSION

GBM is an infiltrative tumor that generates satellite tumors. These are difficult to detect and impede their surgical resection [25]. The prognosis of GBM is very poor. Many studies have shown recently that there is a subpopulation of cells that display stem cell properties in malignant glioma. These cells, which are termed GSCs [26-28], are often resistant to conventional chemotherapy and radiation therapy. They are considered to be the main reason for the origin, migration and recurrence of GBM [29-31]. Thus, new strategies are necessary to reach and eliminate GSCs.

In our study, we isolated hGSCs from primary GBM tissues and identified the characteristics of GSCs by clonal formation assay (self-renew ability), FACS analysis (90% cells in the tumor spheres were double positive on CD133 and Nestin), GFAP and Tuj-1 immunofluorescence experiment (multi-differentiation ability) (Figure 1B). We also performed a serial adoptive transfer assay to identify the serial tumorigenicity of hGSCs. The result showed that 1×10^8^ of these hGSCs could recapitulate the heterogeneity of GBM in nude mice. Furthermore, as Figure 5A indicates, the xenograftes are highly invasive (H&E staining) and glia cell differentiation (GFAP positive) and highly proliferative (high expression of Ki67). Therefore, we concluded that the tumor sphere that was isolated from primary glioma tissues probably had self-renew ability, multi-differentiation potential and a serial tumorigenicity, so that they are GSCs enriched.

NSCs have shown a preferential homing capacity in response to glioma pathophysiology and microenvironment [13]. Stem cells represent a delivery system that can respond to the diverse pathological signals of proliferating tumors. The tropism of hNSCs toward intracerebral tumors suggests a possibility of using them to transport therapeutic molecules to tumors [32-35]. Studies have revealed several factors that are involved in the mechanisms of NSC migration to glioma, including hypoxia and chemokines [36-39]. However, the mechanism that underlies hNSCs tropism to hGSCs is not completely understood. In order to understand this mechanism better, we believe that it is important to distinguish genetic differences between hNSCs and hGSCs. In our previous study [24], we found several essential signaling molecules that were significantly up-regulated in hGSCs in comparison to hNSCs. These signaling molecules may be involved in the hNSCs tropsim to hGSCs (Table 1). They are related to cell migration and adhesion (MIEN1, ICAM1, MMP9, and ANXA2), Neural stem cells growth (NRN1, LIF, and EGFR) and inflammatory reaction (IL8 and NF-κB) respectively. We believe that the synergistic effects of these factors play main roles in causing hNSCs to migrate to hGSCs. Our finding also provides important clues for future study of the mechanism of the tropsim of hNSCs to hGSCs.

**Table 1:** The 10 up-regulated genes that might be related to the hNSCs tropsim to hGSCs in hGSCs

Gene symbol	Gene description	FC of GSC/NSC	P-value
IGFBP3	insulin-like growth factor binding protein 3	1354.400365	1.54E-05
IL-8	interleukin 8	58.4493428	1.94E-04
MMP9	matrix metallopeptidase 9 (gelatinase B, 92kDa gelatinase, 92kDa type IV collagenase)	49.1709356	2.22E-05
ICAM1	intercellular adhesion molecule 1	16.14517524	2.31E-06
MIEN1	migration and invasion enhancer 1	11.64088456	1.00E-04
NRN1	neuritin 1	9.074899613	8.54E-05
LIF	leukemia inhibitory factor (cholinergic differentiation factor)	8.901819804	1.88E-06
NFKB1	nuclear factor of kappa light polypeptide gene enhancer in B-cells 1	6.137988602	5.01E-05
EGFR	epidermal growth factor receptor	4.434412345	9.67E-04
ANXA2	annexin A2	3.612371009	9.27E-04

A gene was defined as differentially expressed when fold change (FC)> 2(up-regulated) or FC<1/2(down-regulated), and a value of *p* < 0.01.

In our previous study [21] and the report by Lee [23], we found that the activation of BMPs/Smad1 signaling induced differentiation and apoptosis of GBM cells and GSCs. It also inhibited the malignant proliferation of tumor cells *in vitro* and *in nude mice*. The main objective of this study is to combine the tropism of hNSCs to hGSCs with the inhibiting effect of BMP4 on hGSCs to investigate the homing behavior and inhibition effects of BMP4-loaded hNSCs on hGSCs *in vitro* and invasive intracranial glioma in the adult rodent brain. It may be noted that we used GFP and RFP to label the color of hGSCs and hNSCs, respectively, in order to easily distinguish these different contexts. After 14 days of intra-cerebral transplantation, the NSCs and NSCs-BMP4 with red fluorescence were found in a GBM xenograft with green fluorescence in the contralateral cerebral hemisphere (Figure 4B). In addition, these engineered hNSCs were able to release BMP4 and induce differentiation and apoptosis, as well as to arrest the growth of hGSCs *in vitro* and *in vivo* (Figure 3, 4, 5).

In our study, the nuclear translocation of phosphorylated Smad1 was observed after 48 hours of BMP4 over-expression. This indicated that the binding of BMP4 and BMPRs (BMPR-II and BMPR-IB) activated Smad1 signaling. We also observed that the effects of NSCs-BMP4/CM on hGSCs were blocked by co-expression of Smad6, which is the specific inhibitor of the BMPs/Smad1 signaling pathway (Figure 3A, 3B). These results indicate that hGSCs are able to respond to BMP4 by phosphorylation of Smad1 and subsequent activation of the BMPs/Smad1 signaling pathway. We also found that Smad6 inhibits BMPs/Smad1 signaling without interfering with receptor-mediated phosphorylation of Smad1 (Supplementary Figure S1). It has been reported that Smad6 specifically competes with Smad4 for binding to receptor-activated Smad1, yielding an apparently inactive Smad1-Smad6 complex. Therefore, Smad6 selectively antagonizes BMPs-activated Smad1 by acting as a Smad4 decoy [40]. This is consistent with our result.

In addition, we did not find that BMP4 over-expression could induce differentiation and apoptosis of hNSCs or inhibit the migration ability of hNSCs to hGSCs *in vitro*, although it did promote the proliferation of hNSCs slightly (Supplementary Figure S2). This might be due to the hNSCs specific elevated expression of BMPR-IA. The latter likely induces the proliferation and maintains the undifferentiated state of neural precursor cells [25, 26]. We found in hGSCs that BMPR-IB expressed prominently and that BMPR-IA expressed faintly (Figure 2C). This could explain the effects of BMP4 on hGSCs in our study (Figure 3, 4, 5). It suggests that BMPs signaling may have different roles in hNSCs and hGSCs through different receptors. As delivery vectors, hNSCs were found to have no anti-tumoral effect by themselves (Figure 5). However, they could migrate to the glioma nests and differentiate into astrocytes (Supplementary Figure S3). Thus, we hypothesized that hNSCs may be able to localize the tumors under physiological conditions so that they could assist tissue repair. However, we observed no malignant transformation of implanted hNSCs, as determined by H&E staining and immunofluorescence assays (Figure 4, 5).

Our study indicates that hNSCs can deliver BMP4 that can be released from hNSCs to achieve tumor inhibitive effects and to extend animal survival. We found that BMP4 overexpression could not affect the preferential homing nature of hNSCs to hGSCs (Figure 4). In fact, because the hNSCs that were overexpressed with BMP4 have a tendency to bind to the BMPRs that expressed on the surface of hGSCs, so the BMP4 overexpression may promote the migration of hNSCs to hGSCs.

Our present study showed that BMP4-loaded hNSCs have clinical potential because they are efficacious in inducing an anti-tumoral effect and tracing hGSCs cells. Because BMP4 is a cytokine that exists in normal CNS, it may be used in the clinic as an adjuvant in a combined therapy against GBM without adding much toxicity to normal brain tissues. Our findings provide further support to the amelioration of safer, more effective, NSC-based gene therapy for malignant glioma.

## MATERIALS AND METHODS

### Isolating and expanding hNSCs and hGSCs

Human NSCs were obtained from the hippocampus of 10-week old fetal tissue of spontaneous abortion. They were cultured *in vitro* by the previously described procedures [24]. Briefly, the dissociated cells were placed into culture plates with a serum-free medium that was supplemented with mitogens (20ng/ml EGF, 20ng/ml b-FGF, and 10ng/ml LIF) (Invitrogen, CA, USA). After seven days, a formation of neurospheres was observed with a phase-contrast microscope. Cells were passaged once a week by 0.025% trypsin and mechanical dissociation and re-plated at a density of 50000 cells/ml. Human GSCs were isolated from three glioblastoma specimens of GBM patients. Tumor nodules were minced and digested by 0.025% trypsin at 37°C for 15 minutes. After filtration through 70 μm mesh, the dispersed cancer cells were collected by centrifugation and then cultured in the same medium that was used for hNSCs above. The culture media were changed every three days. Tumor spheres of different passages were dissociated into single cells that were subsequently plated with one cell per well. The formation of tumor spheres was observed with use of a phase-contrast microscope.

### Ethics statement

Informed consent was obtained from all participating patients, who understood the purpose and risk of providing specimens. This study was approved by the Medical Ethics Committee of Navy General Hospital, PLA, Beijing, China (Permission number: 0506-2006). It was conducted in strict accordance with the recommendations in the guide for the care and use of laboratory animals of the Chinese Institutes of Health. The protocol was approved by the Committee on the Ethics of Animal Experiments of Navy General Hospital (Permission number: 0308-2013). All surgeries were performed under sodium pentobarbital anesthesia, and every effort was made to minimize suffering.

### Vectors construction and cell transduction

To independently identify implanted cells and observe the migration of hNSCs to the xenografted tumor *in vivo*, the hNSCs were infected with a lentivirus to express red fluorescent protein (RFP) and the hGSCs were infected with a lentivirus to express green fluorescent protein (GFP). We constructed another lentiviral vector to express BMP4. The virus was obtained according to the manufacturer's instructions (Life Technologies, USA). A complementary human DNA (cDNA) BMP4 fragment was directionally cloned into the PDC316 plasmid. This plasmid was subsequently recombined with the pLenti6.3 vector, which has the sequences that are required to construct the lentivirus. Then, the pLenti6.3-BMP4 plasmid was transfected with the pLP1, pLP2 and pLP/VSVG plasmids into 293 cells. After 48 hours.. the virus was harvested from the culture medium and filtered. The lentivirus with the BMP4 sequence was obtained according to the manufacturer's instructions. To generate the RFP/BMP4-NSC, the following protocol was followed. RFP-NSCs were infected with the lentivirus that contained the BMP4 sequence (MOI, 50 vp/ml) and then selected after two weeks using G418. Thus, these cells constitutively express RFP and BMP4. The pCS2/Smad6-EGFP expression plasmids were kindly provided by Ye-guang Chen (Tsinghua University, Beijing, China). Transfection was accomplished by using Lipofectamine 2000 reagent (Invitrogen, USA) according to the manufacturer's instructions. The transfection efficiency was greater than 80%, (Supplementary Figure S4).

### ELISA assay for BMP4

Culture supernatants were harvested and used to measure the extra cellular concentration of BMP4 by a commercial, sensitive, enzyme-linked immuno sorbent assay (ELISA) kit (AssayMax; AssayPro LLC, Winfield, MO). The total protein content of cell monolayers was estimated by a Pierce BCA protein assay kit (Thermo Scientific, Rockford, IL, USA) and used to normalize the results.

### Western blot analysis

The isolation of proteins from infected cells, non-treated cells and tumor cells was described previously [24]. Briefly, total protein was extracted by using NP40 lysis buffer (0.5% NP40, 250mM NaCl, 50mM HEPES, 5m Methylene diamine tetra-acetic acid, 0.5m Megtazic acid). It was supplemented with protease inhibitor cocktails (Sigma-Aldrich, St. Louis, Missouri). The lysates were centrifuged at 12,000 rpm for 10 mins and the supernatant was collected for use in experiments. Protein lysates (40 mg) were resolved on denaturing sodium dodecyl sulfate polyacrylamide gels that ranged from 4% to 20% and transferred to nitrocellulose membranes (Bio-Rad Laboratories, Hercules, CA). The membranes were probed with the following antibodies: Rabbit anti-human BMP4 polyclonal antibody (Abcam, UK) and Mouse anti-human CD133 polyclonal antibody, BMPR-IA, BMPR-IB and BMPR-II antibodies (Abcam, UK) and goat anti-Smad1 antibody and rabbit anti-phospho-Smad1 antibody (Cell signaling, USA). Then, the secondary antibodies, which were labeled by horseradish peroxidase, were added and visualized using an ECL chemiluminescent reagent kit (Amersham GE Healthcare, USA).

### Immunofluorescent staining

Cells were washed three times with ice-cold PBS and fixed with 4% paraformaldehyde-PBS. After 15 min of incubation with 0.1% Triton-PBS, the cells were blocked with 1% bovine serum albumin-PBS. Then, the following primary antibodies were used: (1) for hNSCs characterization, mouse anti-nestin polyclonal (1:1000, Santa Cruze, USA); (2) for hNSCs differentiation, the primary antibodies including Rabbit anti-glial fibrillary acidic protein (GFAP) (1:1000, Santa Cruze, USA), Rabbit anti-oligophrenin-4 (O4) (1:1000, Santa Cruze, USA), and mouse anti-Tuj-1(1:1000, Santa Cruze, USA) were used; (3) for hGSCs characterization, the hGSCs sphere were then incubated with mouse anti-human CD133 polyclonal antibody and mouse anti-nestin polyclonal (1:1000, Santa Cruze, USA); (4) for detection of the expressions of BMPs signaling molecules, mouse anti-human BMPR-IA, BMPR-IB and BMPR-II (Abcam, UK) were used, as well as mouse anti-Smad1 antibody, rabbit anti-phospho-Smad1 antibody (Cell signaling, USA); and (5) for examination of the proliferation and differentiation of hGSCs, mouse anti-Ki67 polyclonal antibody and mouse anti-GFAP monoclonal antibody (Abcam, UK) were used.

### Cell proliferation, apoptosis and migration

Cell proliferation was examined by means of a CCK-8 assay. Then, hGSCs were placed onto a 96-well plate with approximately 2×10^3^ cells per well. After the hGSCs were co-cultured with Con/CM, NSCs/CM and NSCs-BMP4/CM for 24, 48 and 72 hours, respectively, the CCK-8 reagent was added to the cells. Then, the cells were further cultured in the chamber for two hr. Next, the optical density (OD) of 450 nm was measured by a microplate reader according to the manufacturer's instructions (EnSpire, PerkinElmer Company, USA).

The apoptotic cell death in tumor spheres and tumor specimens of the nude xenografted model were examined by the TUNEL method using an *in situ* cell death kit (Roche, Indianapolis, IN, USA). We conducted the experiment according to the manufacturer's instructions. The reaction mixture was incubated without enzyme in a control cover slip to detect nonspecific staining. DAB substrate was used to convert the fluorescence signal. Then, the positive cells were examined under a laser-scanning confocal microscopy and a light microscope (Olympus BX-51). Twenty fields in three independent experiments were counted.

The tropism of hNSCs for tumor cells was determined by an *in vitro* migration assay, according to the method of our previous study [41]. Transwell invasion assay was used to determine the specific tropism of NSCs and NSCs-BMP4 to hGSCs. Normal human astrocytes (NHAs), hNSCs-RFP and hNSCs-RFP/BMP4 in serum-free medium were placed in the upper wells of 24 mm tissue culture Trans-well plates (12Am, Nunc, Naperville, IL) that were coated with poly lysine and Matrigel (1 mg/mL in a-MEM), while hGSCs that had been incubated in serum-free medium, were placed into the lower wells of the Trans-well plates and incubated for 48 hours at 37°C. The migration ratio was determined by fixing the membrane, staining the cells using crystal violet 0.1% (Amreco, USA), directly counting the number of migrated cells in 10 high-power fields, and calculating the mean. All experiments were performed in triplicate.

The migration ability of hGSCs was determined by a wound scraping assay. hGSCs that were co-cultured with indicated CMs, as indicated previously, were grown in 6-well culture plates that contained DMEM with 10% FBS. After the cells reached a 90% confluence, the medium was replaced by FBS-free medium for 24 hours. A sterile 200-μl pipette tip was used to create a wound in the monolayer by scraping. The cells were washed with PBS and grown in a FBS-free medium for 24h. The wounds were observed under a phase contrast microscope (Olympus, BX2). The width of scratch was measured at 0 and 24 hour following treatment. The migration rates of the tumor cells were calculated according to the following formula: (cell-free area at the 0 hour cell-free area at the 24th hour)/cell-free area at the 0 hour. Experiments were conducted three times in duplicate and produced comparable results. The values are shown as mean ± SD.

### Intracranial implantation of hGSCs and hNSCs in nude mice

Female nude mice (nu/nu, 25-28 g in weight) were used to conduct experiments *in vivo*. To examine the *in vivo* effect of hNSCs-BMP4 on hGSC, we conducted an intracerebral xenografted tumor experiment and subcutaneous xenografted tumor experiment. Nude mice were anesthetized and stereotaxically inoculated in the right striatum (Bregmaanteroposterior: −0.5mm, mediolateral: +2mm, dorsoventral: −3mm) [41] with 1×10^8^ hGSCs-GFP. Seven days after intracerebral tumor implantation, the mice were divided into three groups (n=10 per group). Group1 was inoculated in the contralateral cerebral hemisphere (Bregma anteroposterior: −0.5mm, mediolateral: −2mm, dorsoventral: −3mm) with 1×10^8^ NHAs-RFP. Groups 2 and 3 were inoculated with 1×10^8^ NSCs-RFP and NSCs-BMP4-RFP, respectively, and also in the left striatum (equivalent position). Subsequently, NHAs, NSCs and NSCs-BMP4 were allowed to migrate for three weeks. Then, the mice were anesthetized deeply and perfused with saline that contained 100U/ml of heparin (Sigma-Aldrich, USA). They were then fixed with 4% of poly-formaldehyde that was prepared in PBS. Migration was assessed. NSCs and hGSCs were identified by means of epifluorescence, and tumor volume was established by determining the volumes of the xenograft in subcutaneous tissue. The paraffin sections (4 μm) of the xenografted tumors were analyzed by H&E staining and immunohistochemical staining.

### Subcutaneously implantation of hGSCs and hNSCs in nude mice

For hGSCs identification, we conducted serial subcutaneous xenografted experiment with the nude mice in which 1×10^8^ hGSCs-GFP was injected into the right armpits of the mice. The tumor growth after two weeks was observed. The generated tumors were then cut out and dissociated into single cells, of which 1×10^8^ cells were then injected into the right armpits of nude mice, as a serial adoptive transfer. The tumor growth after another two weeks was monitored and prepared for another adoptive transfer. The serial adoptive transfer was performed eight times. We observed tumor formation after two weeks of hGSCs transplantation each time.

To study the kinetics of glioma cells growth *in vivo*, the hGSCs (1×10^8^ cells) that were treated with the CM of NHAs, hNSCs and hNSCs-BMP4 were injected s.c. into the right armpit of nude mice. Then, 30 days following injection, the xenograft were stripped out and the volumes were measured using the formula: V (mm^3^)=(a)×(b^2^/2), where a is longest tumor diameter and b is the shortest tumor diameter.

### Immunohistochemistry

Nude mice were euthanized, their brains were perfused and dissected as previously described [21], and 30-μm coronal sections were obtained. Free-floating sections of the brains from each group were washed three times with PBS and incubated at room temperature for 1 hour in PBS/Triton X-100 that contained 1% bovine serum albumin (BSA). Then, the sections were treated overnight with the primary antibodies of mouse anti human Ki67 and GFAP monoclonal antibody (Abcam, UK). After treatment with secondary biotinylated antibodies, color reactions were achieved with diaminobenzidine (DAB) (Sigma, USA) and counterstained with Mayer's hematoxylin.

### Statistical analyses

The statistical analyses involved the use of Student's two-tailed test. All statistical analyses and graphics were conducted using the software package SPSS 12.0 (Windows platform). For the survival curves, we used the log-rank test (Mantel-cox) for independent and unbalanced groups. A *P*-value of less than 0.05 was considered to be statistically significant.

## SUPPLEMENTARY FIGURES



## References

[1] Stupp R, Mason WP, Van den Bent MJ, Weller M, Fisher B, Taphoorn MJ, Belanger K, Brandes AA, Marosi C, Bogdahn U, Curschmann J, Janzer RC, Ludwin SK (2005). Radiotherapy plus concomitant and adjuvant temozolomide for glioblastoma. The New England journal of medicine.

[2] Louis DN, Ohgaki H, Wiestler OD, Cavenee WK, Burger PC, Jouvet A, Scheithauer BW, Kleihues P (2007). The 2007 WHO classification of tumours of the central nervous system. Acta neuropathologica.

[3] Giese A (2003). Glioma invasion—pattern of dissemination by mechanisms of invasion and surgical intervention, pattern of gene expression and its regulatory control by tumor suppressor p53 and proto-oncogene ETS-1. Acta Neurochirurgica (Suppl).

[4] Kaluzova M, Bouras A, Machaidze R, Hadjipanayis CG (2015). Targeted therapy of glioblastoma stem-like cells and tumor non-stem cells using cetuximab-conjugated iron-oxide nanoparticles. Oncotarget.

[5] Karpel-Massier G, Ba M, Shu C, Halatsch ME, Westhoff MA, Bruce JN, Canoll P, Siegelin MD (2015). TIC10/ONC201 synergizes with Bcl-2/Bel-xL inhibition in glioblastoma by suppression of Mcl-1 and its binding partners in vitro and in vivo. Oncotarget.

[6] Zhou W, Cheng L, Shi Y, Ke SQ, Huang Z, Fang X, Chu CW, Xie Q, Bian XW, Rich JN, Bao S (2015). Aresenic trioxide disrupts glioma stem cells via promoting PML degradation to inhibit tumor growth. Oncotarget.

[7] Bastien JI, McNeill KA, Fine HA (2015). Molecular characterizations of glioblastoma, targeted therapy, and clinical results to date. Cancer.

[8] Kane JR, Miska J, Young JS, Kanojia D, Kim JW, Lesniak MS (2015). Sui generis: gene therapy and delivery systems for the treatment of glioblastoma. Neuro-Oncology.

[9] Siemens DR, Austin JC, Hedican SP, Tartaglia J, Ratliff TL (2000). Viral vector delivery in solid-state vehicles: gene expression in a murine prostate cancer model. Journal of the National Cancer Institute.

[10] Chatenoud L, Waldmann H (2012). CD3 monoclonal antibodies: a first step towards operational immune tolerance in the clinic. The Review of diabetic Studies.

[11] Maciejewski JP, Sloand EM, Nunez O, Boss C, Young NS (2003). Recombinant humanized anti-IL-2 receptor antibody (daclizumab) produces responses in patients with moderate aplastic anemia. Blood.

[12] Yi BR, Kim SU, Choi KC (2014). Co-treatment with therapeutic neural stem cells expressing carboxyl esterase and CPT-11 inhibit growth of primary and metastatic lung cancers in mice. Oncotarget.

[13] Tyler MA, Ulasov IV, Sonabend AM, Nandi S, Han Y, Marier S, Roth J, Lesniak MS (2009). Neural stem cells target intracranial glioma to deliver an oncolytic adenovirus in vivo. Gene therapy.

[14] Teng J, Hejazi S, Badr CE, Tannous BA (2014). Systemic anticancer neural stem cells in combination with a cardiac glycoside for glioblastoma therapy. Stem cells.

[15] Yamazoe T, Koizumi S, Yamasaki T, Amano S, Tokuyama T, Namba H (2015). Potent tumor tropism of induced pluripotent stem cells and induced pluripotent stem cell-derived neural stem cells in the mouse intracerebral glioma model. International journal of oncology.

[16] Ehtesham M, Kabos P, Gutierrez MA, Chung NH, Griffith TS, Black KL, Yu JS (2002). Induction of glioblastoma apoptosis using neural stem cell-mediated delivery of tumor necrosis factor-related apoptosis-inducing ligand. Cancer research.

[17] Ehtesham M, Kabos P, Kabosova A, Neuman T, Black KL, Yu JS (2002). The use of interleukin 12-secreting neural stem cells for the treatment of intracranial glioma. Cancer research.

[18] Nakano I, Saiqusa K, Kornblum HI (2008). BMPing off glioma stem cells. Cancer Cell.

[19] Panchision DM, McKay RD (2002). The control of neural stem cells by morphogenic signals. Current opinion in genetic & development.

[20] Shuang L, Zengmin T, Feng Y, Peng Z, Yanri W, Xuefeng D, Haitao W, Xiaozhong P, Jiangang Y, Boqin Q, Wenhong F, Ming F (2009). Expression and functional roles of smad1 and BMPR-IB in glioma development. Cancer investigation.

[21] Liu S, Yin F, Fan W, Wang S, Guo XR, Zhang JN, Tian ZM, Fan M (2012). Over-expression of BMPR-IB reduces the malignancy of glioblastoma cells by upregulation of p21 and p27Kip1. Journal of experimental & clinical cancer research.

[22] Piccirillo SGM, Reynolds BA, Zanetti N, Lamorte G, Binda E, Broggi G, Brem H, Olivi A, Dimeco F, Vescovi AL (2006). Bone morphogenetic proteins inhibit the tumorigenic potential of human brain tumour-initiating cells. Nature.

[23] Lee J, Son MJ, Woolard K, Donin NM, Li A, Cheng CH, Kotliarova S, Kotliarov Y, Walling J, Ahn S, Kim M, Totonchy M (2008). Epigenetic-mediated dysfunction of the bone morphogenetic protein pathway inhibits differentiation of glioblastoma-initiating cells. Cancer Cell.

[24] Liu S, Yin F, Zhang J, Whicha MS, Chang AE, Fan W, Chen L, Fan M, Li Q (2014). Regulatory roles of miRNA in the human neural stem cell transformation to glioma stem cells. Journal of cellular biochemistry.

[25] Mira H, Andreu Z, Suh H, Lie DC, Jessberger S, Consiqlio A, San Emeterio J, Hortigȕela R, Marques-Torrejon MA, Nakashima K, Colak D, Gotz M, Farinas I, Gage FH (2010). Signaling through BMPR-IA regulates quiescence and long-term activity of neural stem cells in adult hippocampus. Cell stem cell.

[26] Panchision DM, Pickel JM, Studer L, Lee SH, Turner PA, Hazel TG, Mckay RD (2001). Sequential actions of BMP receptors control neural precursor cell production and fate. Genes development.

[27] Joseph JV, Roosmalen IA, Busschers E, Tomar T, Conroy S, Eggens-Meijer E, Peñaranda Fajardo N, Pore MM, Balasubramanyian V, Wagemakers M, Copray S, Dunnen WF, Kruyt FA (2015). Serum-induced differentiation of glioblastoma neurospheres leads to enhanced migration/invasion capacity that is associated with increased MMP9. PLoS One.

[28] Roester R (2014). Stem cells and the origin of different glioma subtypes. Journal of neurosurgery.

[29] Yin CL, Lv SQ, Chen XY, Guo H (2014). The role of glioma stem cells in gliomatumorigenesis. The journal of frontiers in bioscience.

[30] Modrek AS, Bayin NS, Placantonakis DG (2014). Brain stem cells as the cell of origin in glioma. World journal of stem cells.

[31] Jackson M, Hassiotou F, Nowak A (2015). Glioblastoma stem-like cells: at the root of tumor recurrence and a therapeutic target. Carcinogenesis.

[32] Lai IC, Shih PH, Yao CJ, Yeh CT, Wang-PJ, Lui TN, Chuang SE, Hu TS, Lai TY, Lai GM (2015). Elimination of cancer stem-like cells and potentiation of temozolomide sensitivity by Honokiol in glioblasomamultiforme cells. PloS one.

[33] Borovski T, Beke P, Van TO, Rodermond HM, Verhoeff JJ, Lascano V, Daalhuisen JB, Medema JP, Sprick MR (2013). Therapy-resistant tumor microvascular endothelial cells contribute to treatment failure in glioblastomamultiforme. Oncogene.

[34] Kim SU (2011). Neural stem cell-based gene therapy for brain tumors. Stem cell reviews.

[35] Kauer TM, Figueiredo JL, Hingtgen S, Shah K (2011). Encapsulated therapeutic stem cells implanted in the tumor resection cavity induce cell death in gliomas. Nature neuroscience.

[36] Bao Z, Zhang C, Yan W, Liu Y, Li M, Zhang W, Jiang T (2013). BMP4, a stong better prognosis predictor, has a subtype preference and cell development association in gliomas. Journal of translational medicine.

[37] Zhang S, Luo X, Wan F, Lei T (2012). The roles of hypoxia-inducible factors in regulating neural stem cells migration to glioma stem cells and determinating their fates. Neurochemical research.

[38] Schulte JD, Srikanth M, Das S, Zhang J, Lathia JD, Yin L, Rich JN, Olson EC, Kessler JA, Chenn A (2013). Cadherin-11 regulates motility in normal cortical neural precursors and glioblastoma. PLoS one.

[39] Lee HK, Finniss S, Cazacu S, Xiang C, Poisson LM, Blumberg PM, Brodie C (2015). RasGRP3 regulates the migration of glioma cells via interaction with Arp3. Oncotarget.

[40] Akiko H, Giorgio L, Joan M, Ali HB (1998). Smad6 inhibits BMP/Smad1 signaling by specifically competing with the Smad4 tumor suppressor. Genes development.

[41] Feng Yin, Jianning Zhang, Shuwei Wang, Chunhui Zhou, Mingming Zhao, Wenhong Fan, Ming Fan, Shuang Liu* (2015). MiR-125a-3p regulates glioma apoptosis and invasion by regulating Nrg1. Plos One.

