# Supplementary material for: The homing and inhibiting effects of hNSCs-BMP4 on human glioma stem cells
Source: Oncotarget. 2016 Feb 18;7(14):17920–31. doi: 10.18632/oncotarget.7472 (PMC4951260; doi:10.18632/oncotarget.7472)
Supplement: Supplementary file 1 [file oncotarget-07-17920-s001.pdf]

SUPPLEMENTARY FIGURES

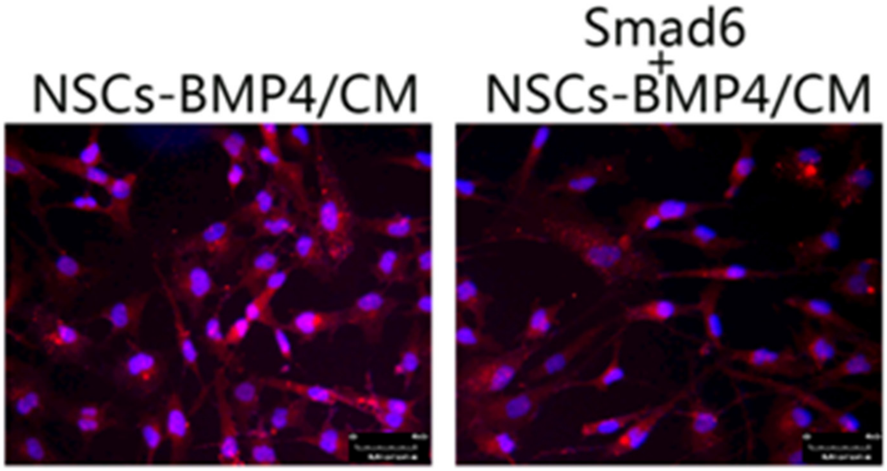

Supplementary Figure S1: Immunofluorescence staining of phosphorylation of Smad1. After transfection of Smad6 for 48hours, significant expression and nuclear translocation of phospho-Smad1 was still observed in hGSCs.

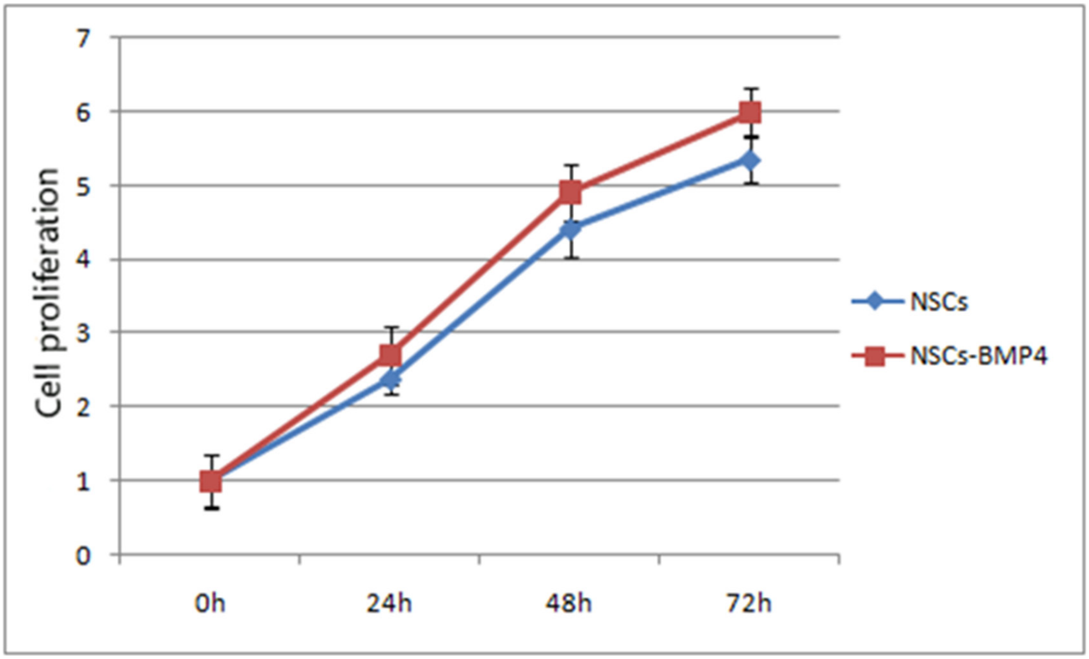

Supplementary Figure S2: The effect of BMP4 over-expression on hNSCs proliferation. The proliferate ability of hNSCs and hNSCs-BMP4 were detected by CCK-8 assay. All experiments were performed in triplicate in three independent sets. The values are shown as means ± SD.

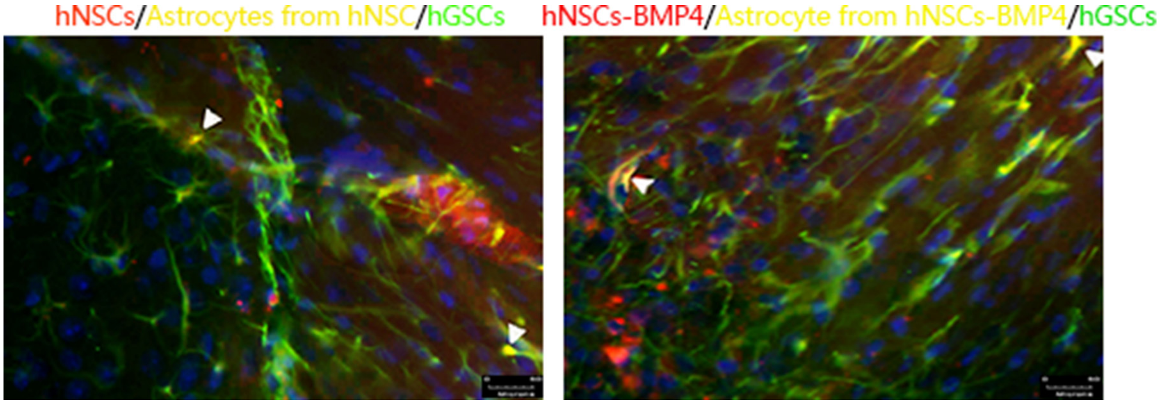

Supplementary Figure S3: hNSCs-RFP and hNSCs-BMP4-RFP were able to migrate into hGSCs-GFP xenograft and differentiate into astrocyte. GFAP immunofluorence staining was undertaken to detect the astrocytes differentiation of hNSCs and hNSCs-BMP4 in the frozen section of xenografts. Using confocal fluorescent microscopy, we observed hGSCs with green color, hNSCs or hNSC-BMP4 with red color and astrocytes that were differentiated from hNSCs and hNSCs-BMP4 with yellow color.

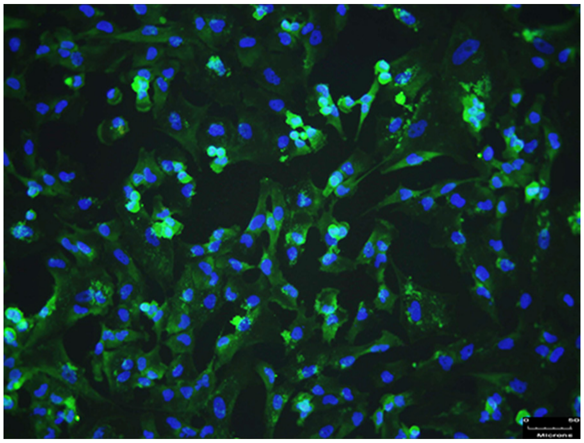

Supplementary Figure S4: The transfection efficiency of pCS2-Smad6. After 48 hours of pCS2-Smad6-EGFP transfection, hGSCs were stained by DAPI and then observed under confocal fluorescent microscopy.
